# Influence of Work-Family Conflict on Turnover Intention of Primary and Secondary School Teachers: Serial Mediating Role of Psychological Contract and Job Satisfaction

**DOI:** 10.3389/fpsyt.2022.869344

**Published:** 2022-04-26

**Authors:** Xiaoyu Li, Xinrui Chen, Dongdong Gao

**Affiliations:** ^1^School of Philosophy and Public Administration, Henan University, Kaifeng, China; ^2^School of Psychology, Institute of Psychology and Behavior, Henan University, Kaifeng, China

**Keywords:** work-family conflict, psychological contract, job satisfaction, turnover intention, teachers

## Abstract

**Objective:**

Based on conservation of resource theory and social exchange theory, to explore how work-family conflict can directly and indirectly influence turnover intention, with psychological contract and job satisfaction as a mediator.

**Methods:**

A total of 505 valid data were collected on primary and secondary school teachers by using work-family conflict questionnaire, turnover intention questionnaire, psychological contract questionnaire and job satisfaction questionnaire from 3 provinces in China. Confirmatory factor analysis was used to evaluate the discriminant validity and common method bias between the four variables through AOMS, the PROCESS macro for SPSS (Model 4 and Model 6) were applied to examine the mediating effect of psychological contract and job satisfaction.

**Results:**

Work-family conflict showed a direct and positive influence on turnover intention; psychological contract was shown to play a mediating role between work-family conflict and turnover intention; job satisfaction was shown to play a mediating role between work-family conflict and turnover intention; and psychological contract and job satisfaction was shown to play a serial mediating role between work-family conflict and turnover intention.

**Conclusion:**

Work-family conflict of primary and secondary school teachers will directly lead to turnover intention. Psychological contract and job satisfaction can reduce the positive influence of work-family conflict on turnover intention. School administrators should help teachers reduce work-family conflict and take effective measures to improve psychological contract and job satisfaction, so as to reduce turnover intention.

## Introduction

It is well-known that work-family conflict inevitably results in a series of negative effects on work (1), one of which is turnover intention (2). Although turnover intention is not actually turnover behavior (3), it weakens job responsibility, reduces job involvement, and decreases job performance (4). For the school, turnover intention means the instability of the teacher team and the decline of the performance, the school should take effective measures to reduce turnover intention. In this study, we will explore how work-family conflict influence turnover intention. The influence process of work-family conflict on turnover intention is complex, which not only has direct influence, but also has indirect influence. This study will take psychological contract and job satisfaction as the mediating variables to explore the indirect influence of work-family conflict on turnover intention.

Psychological contract refers to the mutual expectation or the subjective agreement between individuals and organizations on mutual responsibility obligations (5, 6). Psychological contract reflects that teacher try to keep a balance between contribution and income with schools. The increase of work-family conflict will lead to the destruction of the balance between contribution and income. Therefore, work-family conflict will negatively influence the psychological contract. Because psychological contract focuses on the reciprocal responsibilities and behaviors in the relationship between teachers and schools, it can be inferred that psychological contract can negatively affect turnover intention. So, we think when work-family conflict positively influences turnover intention, psychological contract can buffer its direct effect.

Job satisfaction is an individual's subjective feeling and evaluation of work (7). Work-family conflict will negatively influence job satisfaction because it brings negative emotions such as trouble and distress to teachers. Teachers with high job satisfaction generally do not have turnover intention, so job satisfaction will negatively influence turnover intention. So, we think better job satisfaction can not only stimulate an individual's positive working state, but also reduce the influence of work-family conflict on turnover intention (8).

**Figure 1:**
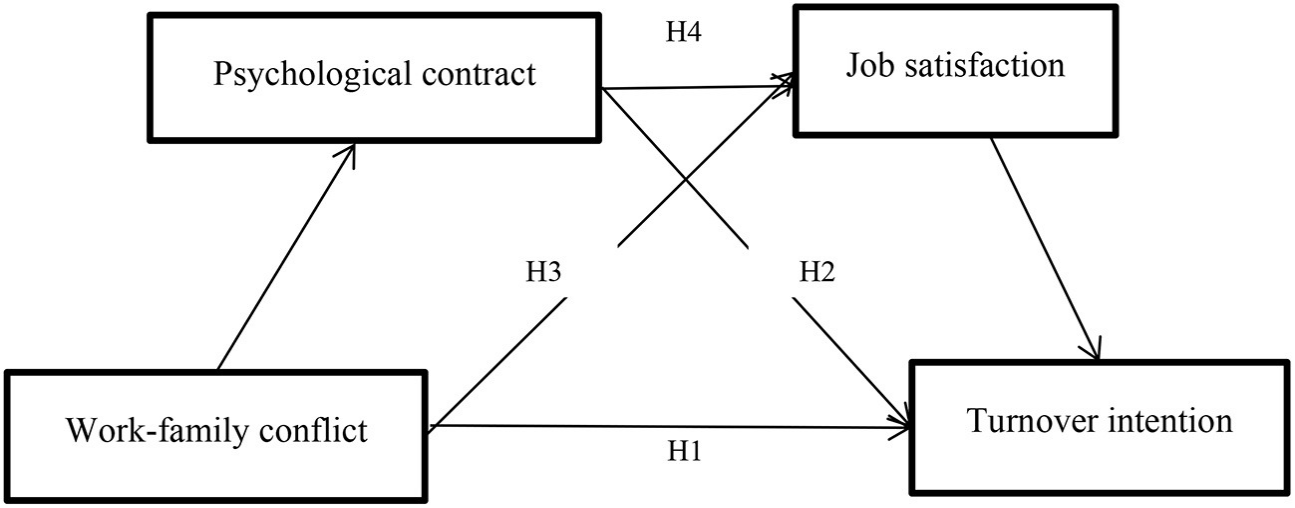
Hypothesized model.

Although the influence impact of work-family conflict on turnover intention has been supported by relevant literature, the research on the mediating effect between work family conflict and turnover intention is not rich enough, especially the research on multiple mediating effect is less. In order to further explore the influence mechanism of work family conflict on turnover intention, this study takes primary and secondary school teachers as participant, takes psychological contract and job satisfaction as mediating variables, and constructs a serial mediated effect model between the four variables, which can not only explore the direct influence of work-family conflict on turnover intention, but also explore the indirect influence of work-family conflict on turnover intention. The research model is shown in Figure 1 and the research questions (RQ) addressed were as follows:

RQ1: How does work-family conflict influence turnover intention?

RQ2: How does psychological contract mediate the relationship between work-family conflict and turnover intention?

RQ3: How does job satisfaction mediate the relationship between work-family conflict and turnover intention?

RQ4: How do psychological contract and job satisfaction play a serial mediating role between work-family conflict and turnover intention?

The remainder of paper is organized as follows. Section Hypotheses Development proposed four research hypotheses. Section Research Methodology describes research method. Section Research Results presents the results of the data analysis. Sections Discussion discussed the research results. Section Limitations and Future Research lists the limitations and future research. Section Conclusion states the conclusions of this study.

## Hypotheses Development

### The Influence of Work-Family Conflict on Turnover Intention

The rapid development of economy and society has resulted in an increase in not only workplace competition but also conflicts between work and family. Since the 1980s, work-family conflict has attracted the attention of scholars, gradually becoming a hot topic of research. Researchers believe that work-family conflict is a form of role conflict. When stress from work adds to the stress from family, or when work roles make family roles more difficult, work-family conflict emerges (9). Work-family conflict has been subdivided into two types: work that affects family (work-family conflict) and family that affects work (family-work conflict). Studies have also shown that among these two types, work-family conflict is more likely to occur (10). A meta-analysis revealed that the impact of work-family conflict is far greater than that of family-work conflict (1). A study on primary and secondary school teachers also proved that the interference of work with family is far greater than that of family with work (11, 12). This study also believes that in the context of Chinese culture, for primary and secondary school teachers, the main impact is work affects family, so it mainly discusses work-family conflict.

Turnover intention refers to the idea of individuals in an organization leaving their current workplace or job. Although it is only an idea rather than an actual behavior, it is an effective indicator that predicts turnover (12). Individuals with turnover intention may cause a series of negative impacts on their organizations (13). The impact of work-family conflict on turnover intention can be explained by the conservation of resource theory, which implies that work-family conflict is caused by the individual's excessive investment and consumption of resources in the work field, resulting in insufficient resources invested in the family field (14, 15). Work-family conflict causes trouble, stress, anxiety, and depression to individuals, and when the conflict reaches a certain level and exceeds individuals' ability to cope, the idea of quitting their job arises. A meta-analysis of turnover intention revealed that the correlation coefficient between work-family conflict and turnover intention is 0.20 (16). Several studies have shown that work-family conflict has a significant positive predictive effect on employee turnover intention (17). In summary, we concluded that work-family conflict plants the idea of leaving the profession among primary and secondary school teachers. Therefore, the following hypothesis is proposed:

H1: Work-family conflict positively influences turnover intention.

### Mediating Effect of Psychological Contract Between Work-Family Conflict and Turnover Intention

Psychological contract is an individual employee's subjective cognition or concept of mutual responsibility and obligation between himself and the organization (18). Although unlike an employment agreement or a labor contract, it does not have a clear legal effect, employees have internal expectations and evaluations of what responsibilities they should assume and how much they should be paid. Work-family conflict refers to the negative impact on personal family life due to the high workload and work pressure. Social exchange theory suggests that when individuals feel that they are giving more and receiving less at work, they will experience psychological distress and a sense of imbalance. In order to seek balance, the individual will reduce the effort they expend in their behavior, thereby also reducing the emotional investment and support for the organization (19). When the school allocates too much work and too many tasks to teachers such that their work interferes with their personal family life or rest, work-family conflict emerges. If teachers feel that their pay is not proportional to their investment, and they feel that the obligations and responsibilities of both sides are out of balance, they will reduce the psychological contract to seek balance. It can be inferred that work-family conflict is negatively correlated with and can negatively predict psychological contract.

Turnover intention is an individual's idea of leaving the job, and it is a kind of withdrawal behavior in the workplace (20). Psychological contract is a “psychological account” of the mutual responsibility between the organization and the individual, and it is the reciprocal behavior of both sides. Therefore, when teachers feel that the school is a responsible organization beneficial to their personal growth and development, their intention to leave is less because psychological contract includes not only teachers' current reciprocal behavior, but also their expectation of future rewards. When teachers subjectively believe that the school fails to fulfill its responsibilities or commitments, turnover intention increases (21). Accordingly, it can be speculated that psychological contract is negatively correlated with and can negatively predict turnover intention. Given that work-family conflict can negatively predict psychological contract, and psychological contract can negatively predict turnover intention, this study proposes the following hypothesis:

H2: Psychological contract plays a mediating role between work-family conflict and turnover intention.

### Mediating Effect of Job Satisfaction Between Work-Family Conflict and Turnover Intention

Job satisfaction refers to the individual's subjective satisfaction with various factors related to work. It is an attitude variable reflecting the individual's positive emotional experience of work (2). All factors related to work can affect job satisfaction. The root cause of work-family conflict is that the work factor affects one's normal family life, which results in various troubles to individuals. According to the social exchange theory, negative attitudes toward work arise and subjective evaluation of work decreases because individuals believe that it is work that causes them unnecessary problems, thereby attributing the cause of the problems to their work (22). Subsequently, job satisfaction will decrease (23). A meta-analysis revealed that work-family conflict has a negative predictive effect on job satisfaction (24). This study suggests that work-family conflict is negatively correlated with and can negatively predict job satisfaction (25, 26).

Turnover intention is a subjective idea generated by individual dissatisfaction with work. When individuals are satisfied with their work, their turnover intention will naturally decrease. A good working atmosphere, humane management system, reasonable salary and benefits, and broad scope for career development will increase primary and secondary school teachers' satisfaction with their jobs. In return, teachers will experience a sense of belonging and trust in schools, thereby reducing their turnover intention. Therefore, this study suggests that job satisfaction is negatively correlated with and can negatively predict turnover intention. Because work-family conflict can negatively predict job satisfaction, and job satisfaction can negatively predict turnover intention, this study proposes the following hypothesis:

H3: Job satisfaction plays a mediating role between work-family conflict and turnover intention.

### Serial Mediating Effect of Psychological Contract and Job Satisfaction Between Work-Family Conflict and Turnover Intention

Both psychological contract and job satisfaction are subjective. When the school is perceived to fulfill its responsibility and obligation, it has fulfilled psychological contract, teachers' job satisfaction will gradually improve. According to the theory of social exchange, the essence of social exchange is reciprocity (27). A well-established psychological contract, in which teachers' internal expectations are respected and met, leads to a sense of belonging to the school and satisfaction with their work, which in turn influences their attitudes and behaviors toward the school (28). It can be inferred that psychological contract can positively predict job satisfaction (29), and job satisfaction playing a mediating role between psychological contract and turnover intention. Because the mediating roles of psychological contract and job satisfaction between work-family conflict and turnover intention have been discussed, respectively, in previous research, the following hypothesis is proposed:

H4: Psychological contract and job satisfaction play a serial mediating role between work-family conflict and turnover intention.

## Research Methodology

### Research Approach

Questionnaire survey was used in this study, which is a popular and extensively used method to collect data from a target population (30). There are two main reasons for adopting this research approach. First, the purpose of this study is to explore the direct and indirect influence of work-family conflict on turnover intention, the questionnaire survey is helpful to measure variables. Second, the questionnaire survey is fast and low cost to collect data, it is commonly and widely used by researchers (31).

### Questionnaire Development

This study aims to explore how work-family conflict direct and indirect influence turnover intention. With psychological contract and job satisfaction as mediating variables, this study constructed a serial mediation effect model. Four questionnaires used in this study were derived from existing literature and have been used several times in published academic articles, showing good reliability and validity. All the questionnaire items were rated on a five-point Likert scale, with 1 implying “completely disagree” and 5 implying “completely agree.” Three academic professors and seven primary and secondary school teachers review the questionnaires and the four questionnaires were regarded as concise and easy to understand. All questionnaires that we used in this study are given in Appendix A in the Supplementary Material.

### Sampling and Data Collection

In this study, 567 questionnaires were distributed in primary and secondary schools in Henan, Anhui, and Jiangsu provinces. Of these, 505 valid questionnaires were recovered, the effective response rate being 89.07%. During the period when we distributed the questionnaire (August 2021), COVID-19 outbreaks occurred in different provinces in China, and primary and secondary schools took strict prevention and control measures against the epidemic. Therefore, questionnaires could only be distributed through the combination of online survey and on-site survey. The network survey was carried out through professional platform what is named “Wenjuanxing,” and the on-site survey was carried out by sending questionnaires to the participants and taking them back after answering them. In order to ensure the data quality, we took three measures: First, select high-quality questionnaires for the survey, and the total number of items in the four questionnaires is 20, so as to avoid boredom or fatigue of the participants. Second, emphasizes that this survey is only for academic research, so the participants should fill in according to their real situation to reduce their defensive and social approval effect. Third, after collecting the questionnaire, review the answers, delete more than 3 missed answers and linear, wavy answers of the questionnaire.

### Variables and Measures

In this study, work-family conflict is used an independent variable, psychological contract and job satisfaction are used as mediating variables, turnover intention is used as dependent variable.

#### Work-Family Conflict Questionnaire

The work-family conflict questionnaire developed by Netemeyer et al. (32), includes 5 items. The sample items include “My work requirements have affected my family life” and “My working hours make it difficult for me to meet my family responsibilities.” In this study, the Cronbach a coefficient of this questionnaire was 0.87.

#### Psychological Contract Questionnaire

The psychological contract questionnaire developed by Liu et al. (33), includes 8 items. The sample items include “I am willing to cooperate with my colleagues” and “I am willing to help my colleagues.” In this study, the Cronbach a coefficient of this questionnaire was 0.77.

#### Job Satisfaction Questionnaire

The job satisfaction questionnaire compiled by Hackman and Oldham (34), translated by Shu and Liang (35), includes 3 items. The sample items include “On the whole, I am satisfied with my work” and “I am generally satisfied with the sense of achievement I get from this job.” In this study, the Cronbach a coefficient of this questionnaire was 0.90.

#### Turnover Intention Questionnaire

The turnover intention questionnaire developed by Weng and Xi (36), which referred to the scale developed by Mobley et al. (37), includes 4 items. The sample items include “I have basically never thought of leaving my current school” and “I often feel bored with my current job and want to change to a new school.” In this study, the Cronbach a coefficient of this questionnaire was 0.74.

The standard value of Cronbach's a is 0.70 and higher (38, 39), the four questionnaires used were above the standard 0.70.

### Demographic

Table 1 presents the demographic characteristics of the sample. A total of 505 participants in this study, included 198 (39.21%) males and 307 (60.79%) females; 184 (36.43%) participants were aged 30 years and below, 268 (53.07%) were aged 31–45 years, and 53 (10.50%) were aged 46 years and above. With regard to their education, 27 (5.34%) participants had a college degree or lower qualification, 305 (60.40%) had an undergraduate degree, and 173 (34.26%) had a master's degree or higher qualification. There were 286 (56.63%) participants with junior titles, 142 (28.12%) with intermediate titles, and 77 (15.25%) with senior titles. With regard to the school site location, 226 (44.75%) participants were from urban areas and 279 (55.25%) were from non-urban areas.

**Table 1:** Demographic information of sample (*N* = 505).

**Measure**	**Item**	***n***	**%**
Gender	Male	198	39.21
	Female	307	60.79
Age	30 years and below	184	36.43
	31–45	268	53.07
	46 years and above	53	10.50
Education	College degree or lower	27	5.34
	Undergraduate degree	305	60.40
	Master's degree or higher	173	34.26
Titles	Junior titles	286	56.63
	Intermediate titles	142	28.12
	Senior titles	77	15.25
School site location	Urban areas	226	44.75
	Non-urban areas	279	55.25

### Data Processing

In SPSS25, descriptive statistics is used to calculate the mean and standard deviation of each variable, correlation analysis is used to calculate the correlation coefficient between the variables (40). Confirmatory factor analysis is used in AMOS25 to evaluate the discriminant validity and common method bias between the four variables (41). Model 4 of PROCESS3.5 macro for SPSS was applied to examine the mediating effect of the data, and Model 6 was applied to examine the serial mediating effect of the data. All variables were standardized in Model 4 and Model 6 before data analyses (42).

## Research Results

### Confirmatory Factor Analysis

The mean and standard deviation of each variable are shown in Table 3, and these data were used to evaluate the discriminant validity and common method bias between the four variables.

In the fitting index of confirmatory factor analysis, if χ^2^/*df* < 3, RMSEA < 0.08, SRMR < 0.08, TLI > 0.90, CFI > 0.90, which proves that the fitting effect of the model is good. In this study, five models were constructed, Table 2 reveals that the fitting index of the “four-factor model” (χ^2^/*df* = 2.97, RMSEA = 0.06, SRMR = 0.06, TLI = 0.93, CFI = 0.94) is best and other four competition models did not meet the basic requirements. This also shows that the discriminant validity between four variable in this study is good.

**Table 2:** Confirmatory factor analysis results.

**Model**	**χ^2^**	***df***	**χ^2^*/df***	**RMSEA**	**SRMR**	**TLI**	**CFI**
Single-factor model	2075.24	170	12.21	0.15	0.13	0.58	0.63
Two-factor model	1746.21	169	10.33	0.14	0.16	0.65	0.69
Three-factor model a	1408.26	167	8.43	0.12	0.16	0.72	0.76
Three-factor model b	825.54	167	4.94	0.09	0.07	0.85	0.87
Four-factor model	486.72	164	2.97	0.06	0.06	0.93	0.94
Four-factor model + CMV	323.08	144	2.24	0.05	0.04	0.95	0.97

*The single factor model combines the four variables into a latent factor; the two-factor model combines work-family conflict and turnover intention as well as psychological contract and job satisfaction into latent factors; the three-factor model a combines work-family conflict and turnover intention into a latent factor, with psychological contract and job satisfaction being independent factors; the three-factor model b considers work-family conflict and turnover intention as independent factors, with psychological contract and job satisfaction being combined into a latent factor; the four-factor model considers the four variables as independent factors; CMV represents common method variance and “+” represents factor combination*.

In order to control the bias effect of common methods, the questionnaire with good reliability and validity is used as the measuring tools. In the test process, the confidentiality of the results and the use of the results for academic research only were emphasized, and some questionnaire items were scored using the reverse scoring method. The common method bias was evaluated by control unmeasured single method- factor approaches (43, 44). Introduced the common method variance (CMV) in confirmatory factor analysis, Table 2 reveals that the fitting degree of the “four-factor model + CMV” model was not significantly improved (ΔRMSEA = 0.01 < 0.05, ΔSRMR = 0.02 < 0.05, Δ TLI = 0.02 < 0.1, Δ CFI = 0.03 < 0.1). Thus, there is no serious common method bias in this data (45).

The composite reliability (CR) of work-family conflict is 0.87, turnover intention is 0.77, psychological contract is 0.79, and job satisfaction is 0.90. The average variance extracted (AVE) of work-family conflict is 0.58, turnover intention is 0.48, psychological contract is 0.36, job satisfaction is 0.76. According to Fornell and Larcker (46) suggested, CR should exceed 0.6, and AVE should exceed 0.5 under ideal condition (47), while 0.36–0.5 are acceptable (46, 48, 49), so the CR and AVE of this study meet the standard.

### Descriptive Statistics and Correlation Analysis

Table 3 summarizes the mean, standard deviation, and correlation coefficient of work-family conflict, psychological contract, job satisfaction, and turnover intention. The mean values of four valuables ranger from 2.46 to 3.78, while the Standard Deviation ranged from 0.51 to 0.85. The results reveal that work-family conflict is significantly positively correlated with turnover intention (*r* = 0.29, *p* < 0.01) and significantly negatively correlated with psychological contract (*r* = −0.12, *p* < 0.01) and job satisfaction (*r* = −0.29, *p* < 0.01). Psychological contract is significantly positively correlated with job satisfaction (*r* = 0.63, *p* < 0.01) and significantly negatively correlated with turnover intention (*r* = −0.50, *p* < 0.01). Job satisfaction is significantly negatively correlated with turnover intention (*r* = −0.65, *p* < 0.01).

**Table 3:** Mean, standard deviation, and correlation analysis results of each variable.

**Variable**	**Mean**	**Standard deviation**	**1**	**2**	**3**	**4**
1 Work-family conflict	3.06	0.83	1			
2 Psychological contract	3.78	0.51	−0.12^**^	1		
3 Job satisfaction	3.62	0.85	−0.29^**^	0.63^**^	1	
4 Turnover intention	2.46	0.73	0.29^**^	−0.50^**^	−0.65^**^	1

** *p < 0.01*.

### Hypothesis Testing

The input method was used for linear regression analysis in SPSS, and the results revealed that work-family conflict could positively predict turnover intention (β = 0.29, *t* = 6.082, *p* < 0.001). Therefore, Hypothesis 1 was verified.

In SPSS, the PROCESS macro was used to select Model 4 provided by Hayes for mediating effect analysis. When psychological contract is a mediating variable, work-family conflict can negatively predict psychological contract (B = −0.12), psychological contract can negatively predict turnover intention (B = −0.47), work-family conflict can positively predict turnover intention (B = 0.23), indirect effect is 0.06 (BootSE = 0.02, BootCI [0.01, 0.10]), the mediating effect accounts for 21% of the total effect, as shown in Table 4, so Hypothesis 2 was also verified.

**Table 4:** Mediating effect test.

	**Coeff**	**se**	***t***	***p***	**LLCI**	**ULCI**
WFC → PC	−0.12	0.04	−2.68	0.01	−0.21	−0.03
PC → TI	−0.47	0.04	−12.64	0.00	−0.55	−0.40
WFC → TI	0.23	0.04	6.25	0.00	0.16	0.31
Total effect	0.29	0.04	6.80	0.00	0.21	0.37
Indirect effect	0.06					
WFC → JS	−0.29	0.04	−6.88	0.00	−0.38	−0.21
JS → TI	−0.62	0.04	−17.61	0.00	−0.69	−0.55
WFC → TI	0.11	0.04	3.10	0.00	0.04	0.18
Total effect	0.29	0.04	6.80	0.00	0.21	0.37
Indirect effect	0.18					

*WFC, work-family conflict; PC, psychological contract; TI, turnover intention; JB, job satisfaction*.

When job satisfaction is a mediating variable, job-family conflict can negatively predict job satisfaction (B = −0.29), job satisfaction can negatively predict turnover intention (B = −0.62), job-family conflict can positively predict turnover intention (B = 0.11), indirect effect is 0.18 (BootSE = 0.03, BootCI [0.12, 0.25]), the mediating effect accounts for 62% of the total effect, as shown in Table 4, so Hypothesis 3 was also verified.

**Figure 2:**
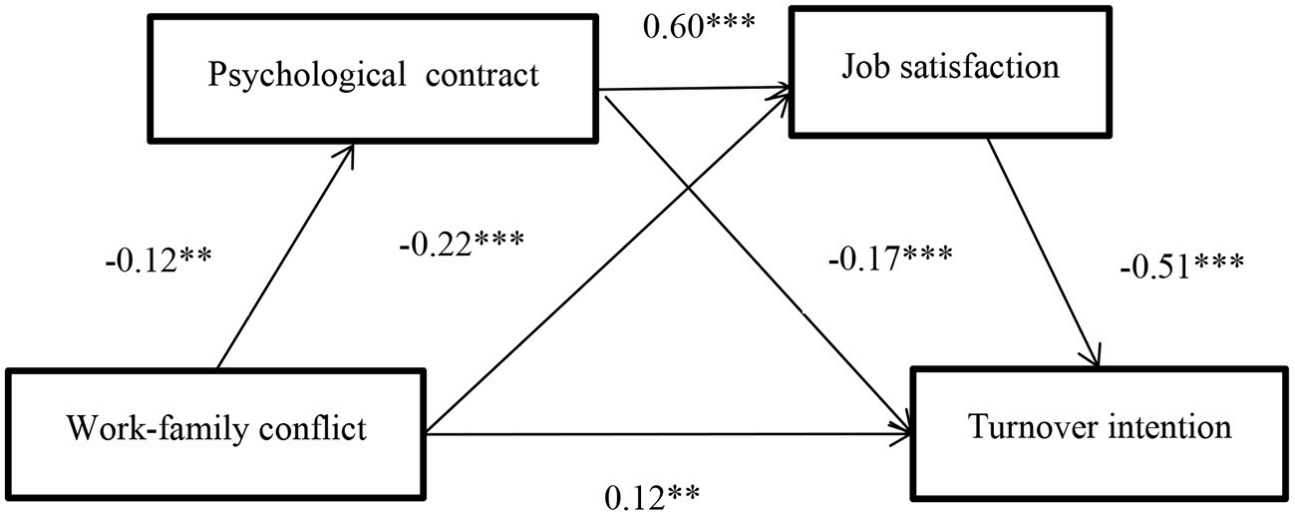
Path analysis of Serial mediating effect. **p < 0.01 and ***p < 0.001.

In SPSS, the PROCESS macro is used to select Model 6 provided by Hayes for serial mediating effect analysis. The results are shown in Figure 2, work-family conflict negatively predicted psychological contract (B = −0.12, *t* = −2.68, *p* < 0.01) and job satisfaction (B = −0.22, *t* = −6.61, *p* < 0.001), psychological contract positively predicted job satisfaction (B = 0.60, *t* = 17.83, *p* < 0.001), and work-family conflict positively predicted turnover intention (B = 0.12, *t* = 3.47, *p* < 0.01). Psychological contract can negatively predict turnover intention (B = −0.17, *t* = −3.94, *p* < 0.001) and job satisfaction can negatively predict turnover intention (B = −0.51, *t* = −11.53, *p* < 0.001). In the whole model, the total effect is 0.29, the direct effect is 0.12, and the total indirect effect is 0.17. There are three mediating paths that are obviously indigenous, as shown in Table 5. Path 1 is work-family conflict → psychological contract → turnover intention, and its effect value is 0.02. Path 2 is work-family conflict → job satisfaction → turnover intention, its effect value is 0.11; path 3 is work-family conflict → psychological contract → job satisfaction → turnover intention, and its effect value is 0.04; as a result, Hypothesis 4 was verified.

**Table 5:** Serial mediating effect test and comparison results of different paths.

	**Effect**	**Boot standard error**	**BootLLCI**	**BootULCI**	**Mediating effect**
Total indirect effect	0.17	0.03	0.108	0.236	58.62%
Path 1 indirect effect	0.02	0.01	0.003	0.044	6.90%
Path 2 indirect effect	0.11	0.02	0.070	0.165	37.93%
Path 3 indirect effect	0.04	0.02	0.007	0.068	13.79%
Comparison 1	−0.09	0.03	−0.153	−0.039	
Comparison 2	−0.02	0.01	−0.044	0.001	
Comparison 3	0.08	0.03	0.026	0.133	

*Comparison 1 is path 1 indirect effect vs. path 2 indirect effect; comparison 2 is path 1 indirect effect vs. path 3 indirect effect; comparison 3 is path 2 indirect effect vs. path 3 indirect effect*.

## Discussion

In Chinese cultural background, both work and family are very important to individuals (12). Chinese people not only pay attention to due diligence and selfless dedication at work, but also pay attention to playing a good role in the family and taking good care of family members (50), which makes them face more conflicts between work and family in their daily life. Chinese have a strong sense of collectivism, in their value system, work is more important than family (2). Therefore, work interferes with family more in China. At this stage, with the introduction of the “double reduction policy” which implies reducing the burden of homework and after-school tutorial classes (51). Society, families, and schools are demanding increasingly more from teachers, who are spending more hours at school fulfilling their additional responsibilities. However, teachers play diverse roles. Besides teaching, many also play a family role (52). The conflict between work and family becomes a problem that primary and secondary school teachers often face (53). Therefore, it is necessary to study work-family conflict and its influence on consequence variables.

This study found that work-family conflict has a positive association with turnover intention, which is the same as most existing research conclusions (16). Primary and secondary school teachers have a special and complex task in teaching primary and secondary school students who are not yet independent. It is necessary to deal with various problems in students' learning and life and to complete other tasks allocated by the school. It is not only a heavy workload but also very trivial (52). Teachers in primary and secondary schools play multiple roles, switching back and forth between work and family, and any disharmony between roles can have a negative impact on teachers' behavior and psychology, inevitably leading to work-family conflict (53). According to the conservation of resource theory, personal resources are limited, if work for a long time consumes resources that are allocated for the family, it will makes teachers feel under pressure and causes dissatisfaction with their work at school (54, 55). Studies have shown that work-family conflict is a source of stress, and they can lead to turnover intention (56, 57). Therefore, work-family conflict can positively affect turnover intention, and the stronger the work-family conflict, the stronger the turnover intention of teachers. This also suggests that we should not only reduce the work burden of teachers, but also improve their work quality and level, so that teachers are able to reduce work-family conflict, so as to reduce turnover intention.

Psychological contract is the subjective cognition and evaluation of mutual responsibilities and obligations between themselves and their schools (18). Work-family conflict is an important source of stress in teachers' work and life (12, 52), when the work-family conflict gradually increases, the role pressure of teachers will also increase (53). They will begin to perceive that school work has an impact on their individual families. Therefore, they believe that the school should bear more responsibilities and obligations, while they should reduce their responsibilities and obligations (58). According to the principle of reciprocity in social exchange theory, it can be inferred that the increase of work-family conflict leads to the decrease of psychological contract (19). Turnover intention is the idea that teachers will leave their jobs or their schools, there are many reasons for turnover intention (59, 60). Psychological contract is an important manifestation of the quality of the exchange relationship between teachers and schools, and it plays a role as a communication bridge between the teachers and schools (12). If there is a good psychological contract between teachers and schools, they will not have turnover intention, and vice versa. When teachers subjectively believe that schools do not take responsibility and fail to fulfill their obligations, which leads to the decline of psychological contract (61). Therefore, psychological contract is negatively correlated with and can negatively predict turnover intention. In view of the relationship between psychological contract and work-family conflict and turnover intention, this study proved that psychological contract plays a mediating role between work-family conflict and turnover intention. In practice, the rights and obligations between schools and teachers should be fixed in the form of system. If the responsibility should be borne by the school, the school should go all out to complete it. If it is the work that should be completed by teachers, the school should make it clear to teachers. When teachers have difficulties, the school should give help in time, so as to form a good psychological contract between the school and teachers.

Job satisfaction is the degree of teachers' satisfaction with the work with which they are engaged (7). When the work-family conflict increases, teachers begin to think that their work affects their families and job satisfaction will decrease. Therefore, work-family conflict can negatively predict job satisfaction (62). Job satisfaction reflects teachers' feelings for the school (63), it is teachers' subjective evaluation of their own work (64), working environment and working treatment. According to social exchange theory, when job satisfaction is high, teachers will have a positive evaluation of the school and generally do not have turnover intention, after all, turnover will bring unpredictable risks, so job satisfaction can negatively predict turnover intention (65, 66). Therefore, job satisfaction plays a mediating role between work-family conflict and turnover intention. In practice, schools should care about teachers' work and life, create a harmonious working atmosphere and good working conditions for teachers, and constantly improve teachers' job satisfaction. When job satisfaction is improved, the impact of work-family conflict on turnover intention will be weakened.

Psychological contract and job satisfaction can play a serial mediating role between work-family conflict and turnover intention because job satisfaction is the result of psychological contract (7). When teachers believe that schools have fulfilled their commitments and personal expectations, their psychological contract will increase, leading to higher job satisfaction. When teachers believe that schools have not fully fulfilled their commitments and failed to meet their personal expectations, their psychological contract will decrease, leading to lower job satisfaction (66). In the whole serial mediating path, according to the social exchange theory (22, 27), when work has a negative impact on the family, teachers will have their own subjective cognition of unequal pay and investment, and the psychological contract will decline or break. This is when the sense of belonging and loyalty to the school will decline, perhaps even causing burnout among individual occupations, resulting in reduced job satisfaction, and subsequent turnover intention (67). This reminds us that psychological contract can improve job satisfaction. Both psychological contract and job satisfaction can buffer the impact of work family conflict on turnover intention. In the process of school management, we should start from the macro and micro aspects, constantly improve the positive factors such as teachers' psychological contract and job satisfaction, and reduce the negative factors such as work family conflict and turnover intention.

This study may have the following implications in theory and practice: First, it is conducive to a deeper understanding of the consequences of work-family conflict and the mediating role of psychological contract and job satisfaction. Second, this study proved that work-family conflict is an important antecedent variable leading to turnover intention and psychological contract and job satisfaction can negatively influence turnover intentions, which enriches the research results of turnover intention. Third, it not only enriches the application situation of the conservation of resource theory and social exchange theory, but also provides a useful reference for understanding the impact of work family conflict on turnover intention. Lastly, school authorities should try and reduce teachers' work-family conflict and increase their psychological contract and job satisfaction, which could consequently reduce teachers' turnover intention.

## Limitations and Future Research

The limitations of this study are mainly manifested in four aspects. First of all, this study is a cross-sectional study, which cannot explain the causal relationship between work-family conflict and turnover intention in a strict sense. A longitudinal or experimental study adopted in future research may reveal more convincing results. Second, this study adopts the convenient sampling method, and the representativeness of the samples is limited; therefore, there is limited generalizability of the results. In the future, more scientific sampling methods can be used to select more samples, so that the results are more universal. Third, among the four questionnaires used, except the psychological contract questionnaire, the other three were not originate in China. Although these three questionnaires were translated by Chinese scholars and have been used for many times in Chinese academic research, cross-cultural use of questionnaires can still lead to bias. In the future, cross-cultural use of questionnaires should be reduced. Finally, this study considers primary and secondary school teachers together. Although both are employees in the field of elementary education, there are some major differences between them. In the future, primary and secondary school teachers should be studied separately for more accurate results.

## Conclusion

Based on the conservation of resource theory and social exchange theory, this study designed a research model to explore the relationship between work-family conflict, turnover intention, psychological contract, and job satisfaction. The result confirmed work-family conflict of primary and secondary school teachers can direct and indirect influence turnover intention. This study revealed that work-family conflict has a direct and positive influence on turnover intention. Psychological contract and job satisfaction can independently mediate the relationship between work-family conflict and turnover intention. The four variables in this study can form a serial mediated effect model. The following conclusions thus arise from the study: First, Work-family conflict is a risk factor of turnover intention, it can directly lead to turnover intention. Second, psychological contract and job satisfaction are the protective factors of turnover intention, they can negatively influence turnover intentions. Lastly, work-family conflict, turnover intention, psychological contract, job satisfaction can form a serial mediated effect model, psychological contract and job satisfaction as mediating variables can reduce the influence of work-family conflict on turnover intention.

## Data Availability Statement

The raw data supporting the conclusions of this article will be made available by the authors, without undue reservation.

## Ethics Statement

The studies involving human participants were reviewed and approved by the Ethics Committee of School of Psychology, Henan University. Written informed consent for participation was not required for this study in accordance with the national legislation and the institutional requirements.

## Author Contributions

XL, XC, and DG designed the study, did the literature review, and wrote the research protocol. XL and XC contributed to the acquisition, interpretation of data, and drafted and revised the manuscript. XL and DG contributed to the revisions in depth for the manuscript. All authors contributed to and approved the final manuscript.

## Conflict of Interest

The authors declare that the research was conducted in the absence of any commercial or financial relationships that could be construed as a potential conflict of interest.

## Publisher's Note

All claims expressed in this article are solely those of the authors and do not necessarily represent those of their affiliated organizations, or those of the publisher, the editors and the reviewers. Any product that may be evaluated in this article, or claim that may be made by its manufacturer, is not guaranteed or endorsed by the publisher.
