# Supplementary material for: Influence of Work-Family Conflict on Turnover Intention of Primary and Secondary School Teachers: Serial Mediating Role of Psychological Contract and Job Satisfaction
Source: Front Psychiatry. 2022 Apr 26;13:869344. doi: 10.3389/fpsyt.2022.869344 (PMC9086593; doi:10.3389/fpsyt.2022.869344)
Supplement: Supplementary file 1 [file Data_Sheet_1.pdf]

Appendix A

Work-Family Conflict Questionnaire

1. My work requirements have affected my family life

2. My working hours make it difficult for me to meet my family responsibilities

3. Because of the work on me, I didn't finish what I wanted to do

4. My work pressure makes it difficult for me to change my family activity plan

5. Because of my job responsibilities, I must make changes to my family activity plan

Psychological Contract Questionnaire

6. I am willing to cooperate with my colleagues

7. I am willing to help my colleagues

8. I play a good exemplary role in my work

9. My work performance is better than the post requirements

10. I am loyal to the school

11. I am loyal to my superiors

12. I will inform the school in advance when I resign

13. I will not support the school's competitors

Job Satisfaction Questionnaire

14. On the whole, I am satisfied with my work

15. I am generally satisfied with the sense of achievement I get from this job

16. I am generally satisfied with my work in this position

Turnover Intention Questionnaire

17. I have basically never thought of leaving my current school

18. I plan to have long-term career development in this school

19. I often feel bored with my current job and want to change to a new school

20. In the next six months, I will probably leave my current school
